# The total hospital and community UK costs of managing patients with relapsed breast cancer

**DOI:** 10.1038/sj.bjc.6604911

**Published:** 2009-02-17

**Authors:** R J Thomas, M Williams, C Marshall, J Glen, M Callam

**Affiliations:** 1 The Primrose Oncology Research Unit, Bedford Hospital NHS Trust, Bedford, MK42 9DJ, UK; 2 Department of Oncology, Addenbrooke's Hospital, Cambridge University NHS Trust, Cambridge CB2 2QQ, UK; 3 Cranfield Health, Cranfield University, Silsoe, Bedfordshire MK43 0AL, UK

**Keywords:** cost, breast cancer, relapse

## Abstract

The complete hospital and community records of 77 women were randomly selected from 232 women who had relapsed breast cancer between 2000 and 2005. Scrutiny of all management activities revealed a total cost of £1 939 329 (mean per patient of £25 186, 95% CI £13 705–£33 821). The median survival from time of relapse was 40.07 months and the median total cost per patient was £31 402.62. Including the community cost of a relapse provides a more realistic figure for future cost-effectiveness analysis of adjuvant breast cancer therapies.

Developments in adjuvant drugs such as herceptin, aromatase inhibitors and taxanes, although initially expensive, reduce the risk of relapse and hence have long-term financial benefits (Thomas *et al*, 2006). To decide whether the benefits are cost-effective, budget holders require an accurate knowledge of how much it costs to treat patients with relapse. Most earlier estimates have used predictive modelling or interviews with clinicians (Mansel *et al*, 2007). A UK trial, now 6 years old, estimated hospital costs of relapsed breast cancer by sending questionnaires to UK oncologists and produced a figure of £12 500 per patient (Remak and Brazil, 2004). A study from Edinburgh, collecting hospital data from node-positive women, gave a figure of £13 533 for loco-regional relapse and £13 193 for distant relapse (Karnon *et al*, 2007). We report the first analysis of the total hospital and community cost of managing patients with relapsed breast cancer from a typical UK breast cancer practice.

## Methods

The Bedford Breast Cancer database identified 232 women who had relapsed between March 2000 and March 2005 with loco-regional or distant disease. The identification codes were placed in separate sealed envelopes and one-third (77) randomly selected for data collection until their death or the predetermined end of the study in January 2007. The average age of the cohort was 62.3 years (33–95) and 44% were originally node positive; 67% post-menopausal, 48% stage 1 (T1), 33% T2, 16% T3–4; 55% oestrogen receptor (ER)^+ve^, 25% ER^−ve^ (25% unknown) and 21% overexpressed human epithelial growth factor receptor (HER)2 (17% unknown). The demographics of the entire group and selected cohort were similar, indicating that the randomisation process was balanced. The specific cancer-related activities were derived from written and electronic hospital records (Table 1). For each patient, a member of the research team visited all GP practices, hospices and community offices to collect all the relapse-related management and drug activities, which ensured there was no missing data. Once complete, the data set was locked and analysed in liaison with the Health Economics Department of Cranfield University. The predetermined subgroups chosen were menopausal status, ER status and HER2 expression, as these influence drug strategies on relapse. Whether patients suffered local or distant relapse was not included, as an earlier analysis did not demonstrate a cost difference (Karnon *et al*, 2007).

The NHS tariffs for outpatient visits, procedures, nights in hospital and investigations were derived from the existing NHS Trust Reference Cost Index 2004 (DoH, 2004). Radiotherapy was costed per fraction taken from the Reference Costs' National Average Unit Cost. General practitioner and district nurse visit from the PCT Reference Cost Index (DoH, 2004). Hospital drug costs included VAT and were the actual amount charged for each. Community drugs (excluding VAT) and hospice (including VAT) drug tariffs were taken from the British National formulary (BNF, 2005).

## Results

**Figure 1:**
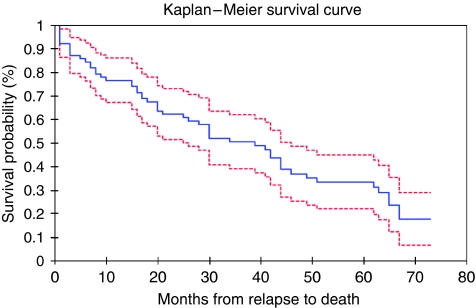
Kaplan–Meier life table and survival curve for 77 patients from date of relapse. Median survival 40.07 months (25 were alive at the time of analysis).

Of the 77 patients analysed in this study, 52 (67.5%) had died of metastatic breast cancer by the end of the data-collection point, January 2007, with a median survival of 40.1 months (Figure 1). The first site of relapse was distant in 51 patients (66%) and loco-regional in 26 (34%). The average time from initial diagnosis to relapse was 71.2 months (range 4–173 months).

The total hospital and community cost of managing the 77 patients from relapse until death, or to the end of the evaluation period, was £1 939 329 (mean £25 186, 95% CI £13 705–£33 821). Dividing the total cost by the total number of patient months within the assessment period (2474.6) gave an average monthly cost of £783.7 per month. The median cost per patient derived from the basic data was £19 886.6. The median cost per patient taking into account that not all patients died during the evaluation period was also estimated by multiplying the monthly average by the median survival (40.07), which gave a figure of £31 402.62.

Nearly a third (30.3%) of the cost of a relapse lay in the community and 69.7% in the hospital setting. The total drug cost was 39% of the total. There was a non-statistically significant trend for patients who were pre-menopausal (32), being subsequently more expensive to manage on relapse than post-menopausal (45) (ratio of mean cost per patient 1.44 : 1). Likewise, ER^−ve^ women were more expensive to treat on relapse than ER^+ve^ (61) (ratio 1.34 : 1). Women with tumours overexpressing HER2 were more expensive to treat than HER2 normal (48) or those not tested (13) (ratio 1.25 : 1). In this study cohort, only 7 of the 16 received herceptin on relapse. There was no trend between those axillary nodes positive (35) compared with those negative (21) or unknown (21) (ratio 1.04 : 1).

## Discussion

The strength of this study lies in the comprehensiveness of the data extracted from a typical breast cancer population treated within a standard UK management framework. Collecting the data from both hospital and community sources required substantial co-operation from a wide range of professional groups, and a single institution ameliorated the absence of missing data. This advantage, however, is also the basis of a potential criticism if this data were to be extrapolated nationally, as a single-institution study may have been subject to skews in local demographics or medical practice.

The demographics of the North Bedfordshire population, obtained from 2003 government figures, however, appeared fairly typical for the United Kingdom (Census, 2001). The average age was in line with the national average as was the single, separated and widowed population. The percentages of people with British citizenship (91.4 *vs* 87.5%) and those born in the United Kingdom (93.0 *vs* 91.1%) were similar. There was a slightly higher percentage of the population working (42.6 *vs* 40.1%), and fewer unemployed (2.6 *vs* 3.4%), but a similar percentage who achieved higher educational qualifications (18.9 *vs* 19.8%). Breast cancer management adhered to West Anglia Cancer Network guidance, which had been developed from national advisory documents. Any deviation from the guidance required formal documentation through a concession form system.

The median overall survival for the patients relapsing at our institution (40.7 months) was higher than the 10–20 months reported in first-line metastatic studies in the United Kingdom and Europe evaluating metastatic chemotherapy regimens (Bontenbal *et al*, 2005), probably explained by our group including 30% with loco-regional relapse only. Overall survival was, however, comparable with the 20–30 months reported within first-line metastatic aromatase *vs* tamoxifen studies (Nabholtz *et al*, 2000).

The median figure of £31 402.6 for treating a relapse is higher than previous UK estimates and this is largely due to the 30.3% of community costs not collected earlier. Even though this data set is the most up to date at the time of publication, these figures represent the last 6 years, and are therefore still likely to underestimate future costs. The full impact of herceptin, for example, was not fully appreciated. Routine use of herceptin in the metastatic setting within our institution was introduced in November 2006, almost halfway through the evaluation period. This meant that only 7 of the 16 HER2-overexpressing patients received herceptin as part of their metastatic management. The average cost of herceptin in treated patients was £15 834.6. According to the original published herceptin metastatic trial data, these patients, on average, could have lived 5 months longer (Vogel *et al*, 2002). In theory, therefore, if the remaining nine patients had received herceptin and each lived 5 months longer, this factor alone would have increased the total cost to over £34 200 per patient.

There is no doubt that newer biological agents, such as oral tyrosine kinase inhibitors and antiangiogenesis drugs, will also substantially add to the metastatic drug costs and as they will keep relapsed patients alive for longer, this will also increase their non-drug costs. The authors intend to repeat the same data-collection exercise every 2 years using the same methodology to give an ongoing estimate of the cost of a relapse, as these newer biological agents evolve into clinical practice. In the mean time, these data by including the community cost improve the accuracy of the cost-effectiveness analysis of adjuvant breast cancer therapies.

## Figures and Tables

**Table 1:** Categories of costs following relapse (77 patients)

	**Source**	**95%**	**Confidence interval**	**Mean**	**Total**
*Community costs (£)*					
GP home visit	GPDB	301	657	480	36 950
GP appointments	GPDB	772	1469	1121	86 337
GP telephone calls	GPDB	57	109	84	6432
Hospice nights	GPBD	495	1796	1146	88 241
Hospice visits	HDB	25	118	72	5554
Palliative community telephone	HBD	39	86	63	4824
Palliative community visits	HDB	272	811	542	41 743
Community district nurse	INR	98	762	430	33 124
GP-prescribed drugs	GPDB	2350	4481	2916	263 036
Hospice drugs	HN	70	614	272	20 954
Sub-total community costs		4479	10 903	7126	£587 195
Percentage total cost (£1 939 329)					30.3%
					
*Hospital drugs*					
Outpatients' hospital pharmacy	PDB	3839	7940	5890	453 495
Ward stores	PDB	47	367	207	15 963
*Hospital bed costs*					
In-patients	PIMS	2166	3802	2984	229 789
Surgical interventions	PIMS	733	1489	1112	85 603
Blood transfusion	HN	372	1044	708	54 534
					
*Outpatients' visits*					
Outpatient consultation	PIMS	2471	3602	3037	233 849
Day/half-day case	PIMS	1040	1981	1511	116 352
ECG	DR HN	41	83	63	4842
Radiotherapy	RTDB	408	764	587	45 166
Support services	DR HN	48	106	78	5981
A and E admissions	PIMS	129	213	171	13 208
					
*Hospital services*					
Radiology	DDB	554	856	705	54 322
Pathology	DDB	292	459	376	28 967
Transport	DR	44	216	131	10 062
Sub-total of hospital costs		9226	22 918	17 560	£1352 134
Percentage of total costs (£1 939 329)					69.7%
					
Grand total		£13 705	£33 821	£25 186	£1 939 329

DDB=departmental database; DR=departmental written records; GPDB=general practice database; HN=hospital notes; INR=individual nursing records; PDB=pharmacy database; PIMS=Patient Information Management System; RTBD=Addenbrooke's oncology electronic database.
